# Duck Migration and Past Influenza A (H5N1) Outbreak Areas

**DOI:** 10.3201/eid1407.071477

**Published:** 2008-07

**Authors:** Nicolas Gaidet, Scott H. Newman, Ward Hagemeijer, Tim Dodman, Julien Cappelle, Saliha Hammoumi, Lorenzo De Simone, John Y. Takekawa

**Affiliations:** * Centre de Coopération Internationale en Recherche Agronomique pour le Développement, Montpellier, France; † Food and Agriculture Organization of the United Nations, Rome, Italy; ‡ Wetlands International, Wageningen, the Netherlands; § US Geological Survey, Vallejo, California, USA

**Keywords:** duck, migration, avian influenza (H5N1), letter

**To the Editor:** In 2005 and 2006, the highly pathogenic avian influenza (HPAI) virus subtype H5N1 rapidly spread from Asia through Europe, the Middle East, and Africa. Waterbirds are considered the natural reservoir of low pathogenic avian influenza viruses (*1*), but their potential role in the spread of HPAI (H5N1), along with legal and illegal poultry and wildlife trade (*2*), is yet to be clarified.

The garganey (*Anas querquedula*) is the most numerous duck migrating between Eurasia and Africa: ≈2 million gather in the wetlands of Western Africa every northern winter (*3*). We report on a spatial correlation between the 2007 migration path of a garganey monitored through satellite telemetry and areas that had major HPAI (H5N1) outbreaks from 2005 through 2007.

Seven garganeys were captured, sampled, and fitted with a 12-g satellite transmitter in northern Nigeria (Hadejia-Nguru Wetlands; 12°48′N; 10°44′E) in the period February 7–15, 2007. All cloacal and tracheal swabs tested negative for avian influenza virus by real-time reverse transcription–PCR analysis of the matrix gene. One second-year (>9-month-old) female garganey migrated from northern Nigeria to Russia in April–May 2007 (Appendix Figure), where she remained until the end of July. During this 6-week spring migration over the Sahara Desert, Mediterranean Sea, and Eastern Europe, this duck stopped at 3 main stopover sites in Crete, Turkey (Bosphorus region), and Romania (Danube River delta). The duck migrated back to the Danube delta in August, where it remained until November, when the signal was lost. Other garganeys we monitored stopped transmitting before initiating spring migration (n = 3) or remained in West Africa during spring and summer (n = 3), which suggests a stress linked to capture or constraint from the transmitter attachment.

This transcontinental migration path connects several areas of past major HPAI (H5N1) outbreaks (Appendix Figure). The wintering area in Nigeria where this duck was caught and remained for 8 weeks before spring migration is located where a large number of outbreaks have occurred repeatedly since February 2006 (the closest being 30 km away). This bird reached its breeding ground in Russia near Moscow and stayed for 2 months in an area that had several outbreaks in backyard poultry in February 2007 (the closest being 30 km away). Finally, the Danube delta, used as a resting ground for 3 months in late summer and autumn, is also an area with recurring outbreaks since October 2005 in wild and domestic birds, with the most recent case reported in November 2007 (the closest being 10 km away). The initial spread of HPAI virus (H5N1) from Eurasia to Africa occurred in autumn and winter 2005–06. The migratory movements we observed during spring and summer in this study were not temporally correlated with any reported HPAI (H5N1) outbreak, either in sequence or period; hence, they should not be interpreted as evidence of the role of wild bird in expansions of the virus.

During spring migration from Nigeria to Russia, the garganey stopped several days in wetlands situated close to areas of past outbreaks in the Danube delta (4 days at a distance of 1–4 km from October 2005 outbreaks) and Lake Kus, Turkey (8 days at a distance of 10–30 km from October 2005 outbreaks). The occurrence of past outbreaks indicates that the duck used wetlands favorable to HPAI virus (H5N1) transmission as stopover sites. The relatively long stopover periods enabled prolonged contact of migratory ducks with local domestic and wild bird populations or through shared water, thus prolonging the potential for virus transmission. Considering the persistence of infectivity of HPAI virus (H5N1) in aquatic habitats (*4*), the number of migratory ducks congregating at stopover sites from various geographic origins and destinations, and the asynchronous timing of the arrival and departure of migratory ducks (*5*), we believe that these sites may provide locations for disease transmission and possible spread upon movement of wild birds.

The satellite-fitted female garganey covered distances between stopover sites of >2,000 km in <2 days, traveling at an estimated speed of 60 km/h. This large-scale movement in a short period, coupled with experimental exposure trials demonstrating viral shedding of up to 4 days in ducks with no clinical signs of infection (*6*), is consistent with potential viral transmission over great distances.

These facts illustrate how a pathogen such as HPAI virus (H5N1) can potentially be transported rapidly by migratory birds across continents. However, the physiologic impact of an HPAI (H5N1) infection on the ability of birds to migrate long distances is still unknown (*7*) and to date, most empirical evidence suggests that wild birds have only moved short distances (a few hundred kilometers) likely carrying HPAI virus (H5N1) (*8*). Despite extensive global wildlife surveillance efforts and with the exception of a few reported cases of HPAI (H5N1) infection in apparently healthy wild ducks (*9*,*10*), evidence of wild bird involvement in the spread of HPAI virus (H5N1) over long distances is still lacking.

## Supplementary Material

Appendix Figure A) Migration route of a garganey tracked with satellite telemetry from February through November 2007, and major highly pathogenic avian influenza (H5N1) outbreaks in domestic and wild birds over Europe, the Middle East, and Africa from July 2005 through November 2007. Close-up maps of stopover sites during the spring migration in (B) the Danube River delta in Romania (May 8-11, 2007), and (C) Lake Kus in Turkey (April 29-May 6, 2007).
