# Supplementary figures and images for: Duck Migration and Past Influenza A (H5N1) Outbreak Areas
Source: Emerg Infect Dis. 2008 Jul;14(7):1164–6. doi: 10.3201/eid1407.071477 (PMC2600360; doi:10.3201/eid1407.071477)

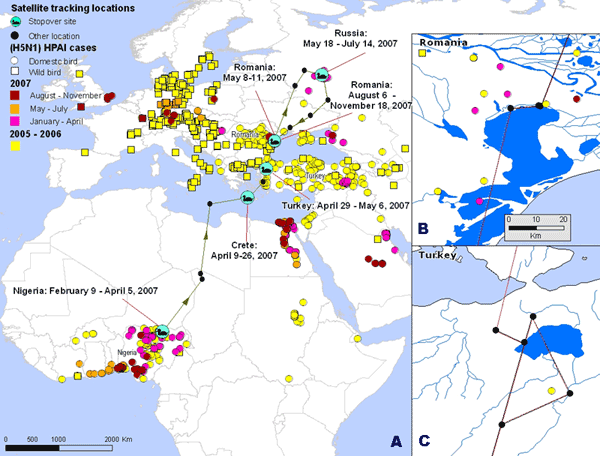

Supplement: Appendix Figure — A) Migration route of a garganey tracked with satellite telemetry from February through November 2007, and major highly pathogenic avian influenza (H5N1) outbreaks in domestic and wild birds over Europe, the Middle East, and Africa from July 2005 through November 2007. Close-up maps of stopover sites during the spring migration in (B) the Danube River delta in Romania (May 8-11, 2007), and (C) Lake Kus in Turkey (April 29-May 6, 2007). [file 07-1477_app-s1.gif]
